# Incidence, mortality, survival, and treatment statistics of cancers in digestive organs—Japanese cancer statistics 2024

**DOI:** 10.1002/ags3.12835

**Published:** 2024-06-17

**Authors:** Takahiro Higashi, Yukinori Kurokawa

**Affiliations:** ^1^ Department of Public Health and Health Policy Graduate School of Medicine, The University of Tokyo Tokyo Japan; ^2^ Department of Gastroenterological Surgery Graduate School of Medicine, Osaka University Osaka Japan

**Keywords:** cancer registries, digestive system neoplasms, statistics

## Abstract

Access to accurate statistical data is paramount in the pursuit of effective cancer control activities, including research, policy development, and clinical care. This paper presents a comprehensive statistical report on the incidence, mortality, survival, and treatment of major digestive organ cancers, including those of the esophagus, stomach, colon, rectum, liver, extrahepatic biliary tract, and pancreas, in Japan. We compiled data from the National Cancer Center's “Cancer Information Services” and government “e‐Stat” websites and offered a succinct overview of basic statistics by using tables and graphical presentations. Our findings underscore the critical role of the National Cancer Registry introduced by the Cancer Registry Act of 2016, which mandates hospitals across Japan to report cancer cases. This system ensures more accurate incidence statistics. Mortality data sourced from the National Vital Statistics System and survival rates derived from hospital‐based cancer registries offer insights into the outcomes and efficacy of treatment modalities. These data indicate a downward trend in mortality for stomach and liver cancers and stable or declining rates for other cancers except pancreatic cancer, which has the lowest survival rate. Treatment patterns indicate an increase in endoscopic procedures for esophageal and stomach cancers, with stable treatment approaches for colorectal cancer. This statistical overview aims to improve the understanding and inform research, policy, and clinical decisions in the field of digestive organ cancers.

## Graphical abstract

Statistical information is essential for clinical cancer research and policymaking processes. The essential information was summarized in this paper to help users of these statistics understand how these are made and what caution they need to take when interpreting the numbers.

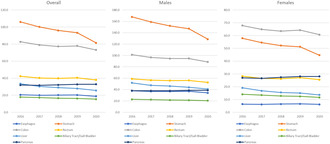

## 1 INTRODUCTION

Accurate statistics are essential for cancer control activities including research, policy, and clinical care.^1^, ^2^, ^3^ However, determining the correct number can be a daunting task for the clinicians. The data are scattered across multiple large tables, and even if we find seemingly correct numbers, it is often challenging to verify the accuracy of the numbers by examining the methodology used to produce them. To apply statistics, we require a clear summary of numbers accompanied by adequate information on the methodology.

To this end, we present the incidence, mortality, survival, and treatment statistics of cancers of the major digestive organs, namely the esophagus, stomach, colon, rectum, liver, extrahepatic biliary tract, and pancreas. Incidence and mortality were adjusted for age and sex to observe the chronological trend in the Japanese standard population in 2015.^4^ The data were obtained from the National Cancer Center's “Cancer Information Services” websites^5^, ^6^ and the Government “e‐Stat” websites,^7^ and they were summarized in tables and graphical presentations. Descriptions of the systems that produced the statistics were derived from the respective government documentation and legal provisions.

## 2 DATA SOURCES FOR EACH STATISTICS

### 2.1 National Cancer Registry (data source for the incidence and treatment statistics)

The incidence and treatment statistics were obtained from the National Cancer Registry.^8^, ^9^ Production of accurate incidence statistics is challenging.^10^ The occurrence of cancer cannot be accurately recorded without mandatory reporting to a central registry. The Cancer Registry Act was enacted in Japan in 2016,^11^ and the National Cancer Registry system was initiated. It mandates all hospitals across Japan to report the basic information about all newly encountered cancer cases (either diagnosed or newly visited after diagnosed elsewhere) to the prefecture in which the hospital resides by the end of the following year. Cases undergo checks for the duplicated reports from multiple hospitals and the duplicated reports detected are consolidated, first at the prefecture level in the prefectural office and then at the national level in the National Cancer Center using personal identifiers such as names, date of birth, and addresses. The data are also supplemented by the death certificate data provided by the Vital Statistics, and the missed reports (i.e., death due to cancer without reported cases) are filled with a follow‐back survey. These processes ensured the accuracy of the incidence statistics. The start of the year 2016 produced an abrupt increase in the incidence of cases at 99 5131^8^ cases compared to 903 914 cases in 2015, which was produced by the summation of prefecture‐operated non‐mandatory registries.^12^ This phenomenon is generally explained by the insufficient consolidation across diagnosis years; a certain proportion of cases reported in 2016 could not be consolidated when they were diagnosed in different hospitals in previous years and were not reported because the reporting mandates had not yet begun.^8^ Such cases remain with uncertain diagnosis year because the cases reported were marked as “diagnosed elsewhere previously” but not consolidated with the report marked as “diagnosed at the reporting facility.” The proportion of such cases decreased gradually from 7.0% in 2016 to 5.0% in 2019.^9^

Another important point in interpreting the registry data is whether the number includes patients with carcinoma in situ. Among the digestive organs, in situ disease is when malignant cells are confined to the epithelium, while there is a different classification of in situ disease when malignant cells are found in the esophagus, colon, and rectum. Often, the numbers in the statistics exclude in situ cases and only include invasive diseases, partially because the International Classification of Diseases, 10th edition, assigns in situ diseases as D codes for benign diseases.^13^ Care must be taken because the number of such cases is usually substantial; approximately 20% of all cases are in situ diseases of the colon and rectum. Users of these statistics must be aware of whether their numbers include in situ cases.

Disease classification in the National Cancer Registry is based on the International Classification of Diseases (ICD) 10th Edition (ICD‐10).^13^ Although the registry uses the ICD‐Oncology 3rd edition (ICD‐O‐3.1),^14^ it was converted to ICD‐10 to maintain consistency with mortality statistics. Therefore, the classification of each cancer type is based solely on the location of the tumor. It does not have a significant effect on most cancers of the digestive organs because the numbers represent the dominant histology. However, we need to be aware that the numbers for liver cancer include all cancers that occur in the liver, including hepatocellular carcinoma and intrahepatic cholangiocarcinoma. The extrahepatic biliary tract includes the papillae of Vater.

### 2.2 National vital statistics

Mortality statistics were obtained from the government's National Vital Statistics System.^15^ When a person dies, a physician issues a death certificate, and the family members (or cohabitants, landlords) are required by law to report the certificate to the Municipal Office within 7 days.^16^ The office marks the person's Resident Registration (*Jumin‐hyo*) and Family Registration (*Koseki*) as dead and sends copies to the National Vital Statistics. The system is considered to function well.^17^ Causes of death have been coded using the ICD‐10 since 1995. The accuracy of the cause of death information is generally good for cancers,^18^ but the detailed interpretation of hepatobiliary cancer may require caution for the reasons described in the ICD‐10.

Notably, the denominator of the mortality statistics was the general population. A common misunderstanding is that mortality can be calculated by subtracting the survival rate from 100%. Because survival rates use a group of patients as the denominator, survival and mortality rates do not add up to 100%.

#### 2.2.1 Hospital‐based cancer registries for survival statistics

Survival statistics were derived from the National Database of Hospital‐Based Cancer Registries^19^ for two reasons: first, the National Cancer Registry is a new system that has not produced survival statistics; second, the National Cancer Registry uses the extent of disease information for statistical purposes instead of clinically used stage information. Although hospital‐based cancer registries do not cover all hospitals in Japan, they cover approximately 70% of all cancer cases in Japan in 2017.^20^ Designated Cancer Care Hospitals traditionally operate these registries. These hospitals are required to function as local centers to provide appropriate cancer treatment and care for residents and to implement cancer control policies and activities in the local healthcare system. The conditions for designation include appropriate equipment, such as radiotherapy apparatus, and staff, such as pathologists, palliative care teams, patient support consultants, and certified cancer registrars. Cancer registrars must receive certification from the National Cancer Center after completing a set of lectures and passing examinations. Certification has two levels, introductory level and standard level. The introductory level covers basic knowledge of cancer control, cancer registries, and the Union for International Cancer Control (UICC) Tumor‐Node‐Metastasis (TNM) classification of five major cancers in Japan, namely, stomach, lung, breast, colorectal, and liver cancers. This standard level covers all other types of cancer. Certifications are granted when they pass the qualifying examination. They are required to renew their certifications every 4 years.

Accurate survival statistics require appropriate patient follow‐ups.^21^ Until 2015 cases, the National Cancer Center referred to the municipal resident registrations maintained by municipalities to confirm the vital status of the patients and provide information to hospital‐based cancer registries. Although some municipalities refused to provide the vital status of the patients owing to privacy concerns, the follow‐up rates were generally high. Since the poor follow‐up rates bias the calculated survival rates, the inclusion rule in the hospital‐based cancer registry survival statistics stipulates that data from hospitals with <90% follow‐up rate (i.e., the vital status of >90% of the patients is known at 5 or 10 years) are excluded.^6^ A total of 425 and 341 hospitals qualified and were thus included in the report for the calculation of 5‐year survival for patients diagnosed with the respective cancers in 2015 and 10‐year survival for patients in 2011, respectively.^6^ For esophageal and colorectal cancers, we included only invasive diseases in the survival calculations. Moreover, we should bear in mind that the stage classifications are based on the UICC system. For liver cancers, the difference from the unique Japanese staging system (*Toriatsukaikiyaku*) may be substantial.

Another point that the users of survival statistics should know is the use of net survival. Survival statistics must consider the possibility that patients may die of causes other than cancer (e.g., cancer patients may die of trauma or heart attack). Net survival is the survival rate adjusted for death from other causes using survival data of the general population in Japan.^22^ The survival rates presented here are net survival rates.

## 3 STATISTICS

### 3.1 Incidence

**FIGURE 1:**
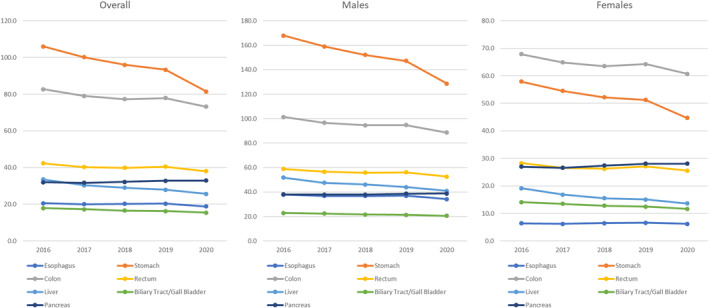
Trend in age‐adjusted incidence of cancers in digestive organs 2016–2020.

The incidence and rate per 100 000 individuals for the respective cancers in 2020 are derived from the and National Cancer Registry report^9^ and presented in Table 1, and their chronological trend after age‐adjustment summarized from the report and Cancer Information Services^5^ is shown in Figure 1. In Table 1, the esophagus, colon, and rectum are presented with both invasive and in situ diseases, showing that 10%, 23%, and 19% were in situ diseases, respectively. The chronological trend after age adjustment for the 2015 Japanese standard population shown in Figure 1 only accounted for trends in invasive diseases. When the colon and rectum were counted separately, the most common cancer in the digestive organs was stomach cancer, but the total number of colorectal cancers exceeded the number of stomach cancers. The decline in incidence for stomach and liver cancers are generally attributable to the decreased prevalence in infection of *Helicobacter pylori* ^23^ and hepatitis viruses,^24^ respectively. Additionally, the decline in age‐adjusted incidence for many cancers in 2020 is due to the decrease in diagnostic opportunities caused by the start of novel coronavirus pandemic.^25^

**TABLE 1:** Incidence of cancers in digestive organs in 2020.

	No. of cases			Incidence (/100 000)		
	Overall	Male	Female	Overall	Male	Female
Esophagus^a^	24 558	20 128	4430	19.5	32.8	6.8
Esophagus (incl. in situ)	27 071	22 116	4955	21.5	36.0	7.6
Stomach	109 679	75 128	34 551	86.9	122.5	53.3
Colon^a^	98 240	51 733	46 507	77.9	84.3	71.8
Colon (incl. in situ)	126 202	69 599	56 603	100.0	113.4	87.4
Rectum^a^	49 485	31 076	18 408	39.2	50.7	28.4
Rectum (incl. in situ)	60 096	38 039	22 056	47.6	62.0	34.0
Liver	34 744	23 707	11 037	27.5	38.6	17.0
Biliary tract	21 392	11 705	9687	17.0	19.1	14.9
Pancreas	44 448	22 557	21 891	35.2	36.8	33.8

^a^ Includes only invasive diseases.

### 3.2 Mortality

**FIGURE 2:**
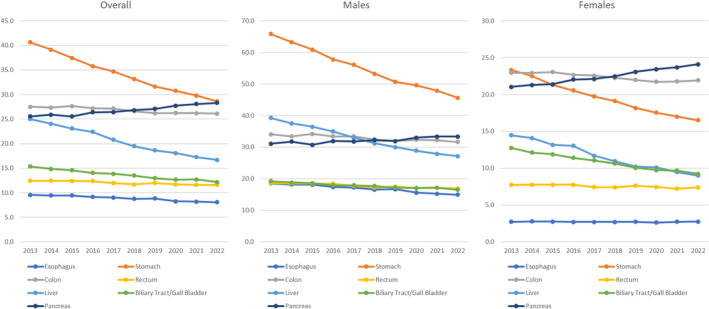
Trend in age‐adjusted mortality of cancers in digestive organs 2013–2022.

Current mortality rates due to cancer of the digestive organs are derived from Cancer Information Services^5^ and e‐Stat^7^ and listed in Table 2 and the time trend of age‐adjusted mortality is shown in Figure 2. Because mortality statistics are based on an established government system, we can follow the long‐term trend of age‐adjusted mortality by eliminating the effect of societal aging. The incidence of stomach and liver cancers is rapidly decreasing in both males and females, and the incidence of most cancers other than pancreatic cancer is stable or gradually decreasing.

**TABLE 2:** Mortality of cancers in digestive organs in 2022.

	No. of cases			Mortality rate (/100 000)		
	Overall	Male	Female	Overall	Male	Female
Esophagus	10 918	8790	2128	8.9	14.8	3.4
Stomach	40 711	26 455	14 256	33.4	44.6	22.7
Colon	37 236	18 215	19 021	30.5	30.7	30.3
Rectum	15 852	9884	5968	13.0	16.7	9.5
Liver	23 620	15 717	7903	19.4	26.5	12.6
Biliary tract	17 756	9470	8286	14.6	16.0	13.2
Pancreas	39 468	19 608	19 860	32.3	33.1	31.7

### 3.3 Survival

**FIGURE 3:**
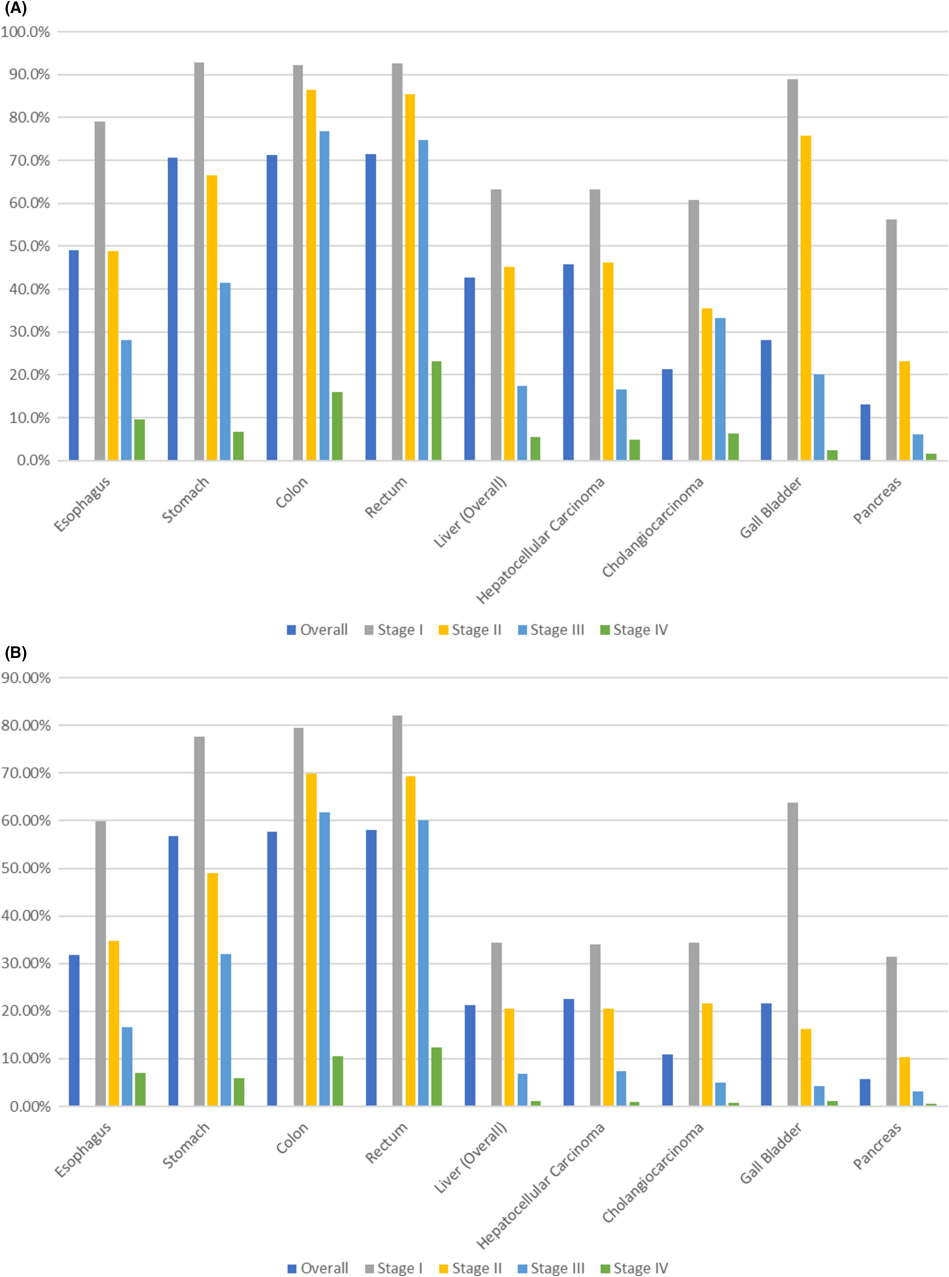
Survival rates of cancers in digestive organs. (A) 5‐year net survival of cases diagnosed in 2015. (B) 10‐year net survival of cases diagnosed in 2011.

Figure 3 shows the 5‐ and 10‐year net survival rates of the respective cancers obtained from reports of hospital‐based cancer registries.^6^ Patients with colon and rectal cancers showed similar survival rates at each stage as well as at overall survival. Because hepatocellular carcinoma and intrahepatic cholangiocarcinoma show similar stage‐specific survival rates, only data for liver cancer are presented. Among cancers of the digestive organs, pancreatic cancers have the worst overall survival at each stage.

### 3.4 Treatment

The first course of treatment for the reported cancers was coded in the National Cancer Registry.^26^ These data were compiled from the e‐Stat website^7^ and included only invasive diseases. The mandated items in the registry include the provision of surgery, endoscopic treatment, chemotherapy, and radiation therapy as part of the first course of treatment(s). First course treatments were defined in the cancer registry as a series of treatments planned immediately after diagnosis. Surgery involves open and laparoscopic surgical procedures. Endoscopic treatment was defined as treatment using optical devices inserted into naturally open foramina, such as the mouth and anus. Chemotherapy also includes immune checkpoint inhibitors and hormone therapy (hormone therapy may not be applicable to the cancer types discussed here). Treatment was recorded only when the treatment aimed to reduce the tumor; therefore, symptom control therapy, such as bypass surgery of the intestine, was not included. Because the treatments provided in different hospitals were consolidated, the data theoretically covered all treatments provided for each case. One case can undergo multiple treatments (e.g., surgery and chemotherapy); thus, the proportions do not add up to 100%.

**FIGURE 4:**
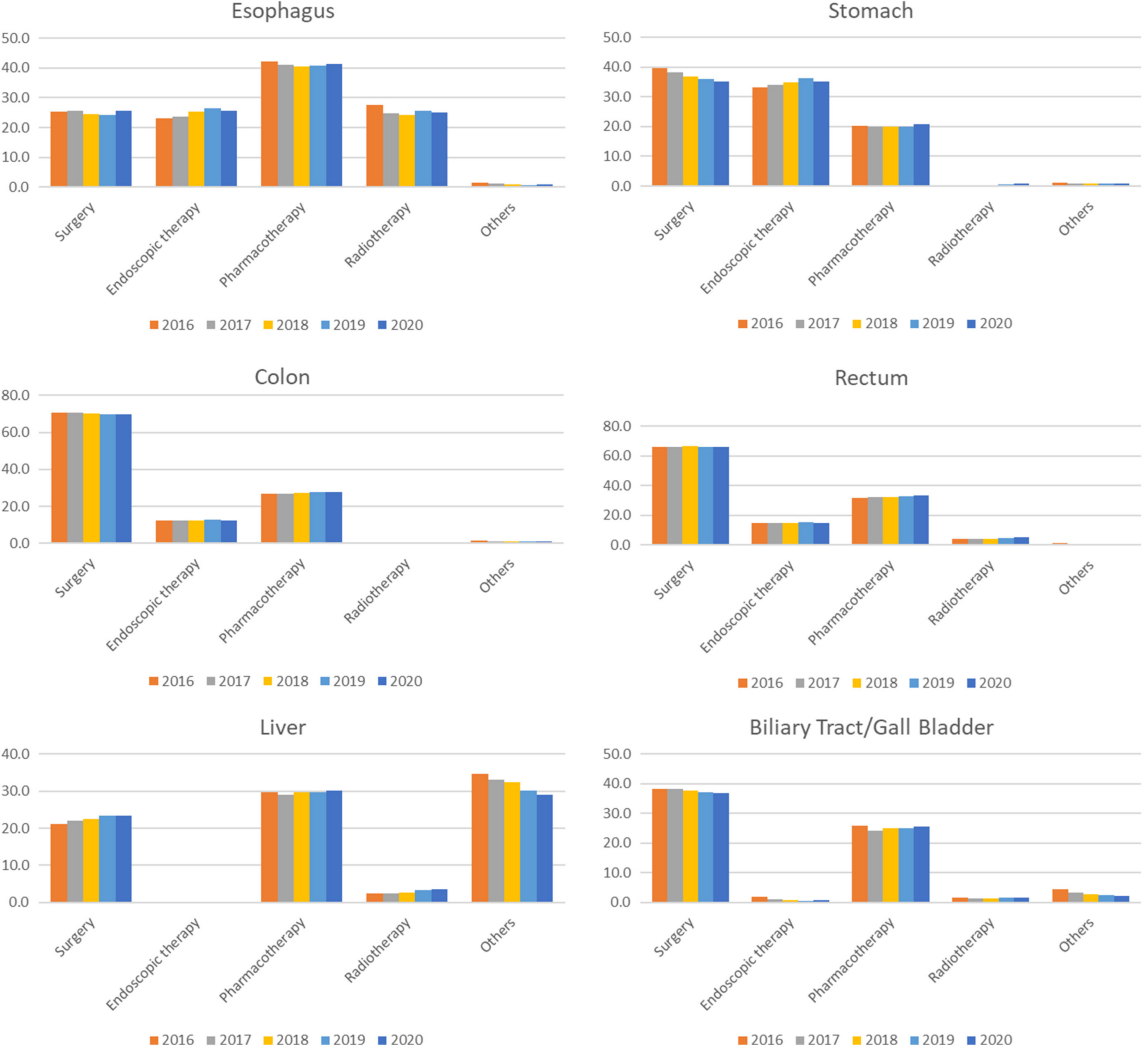
Trend in the treatment of cancers in digestive organs 2016–2020.

Figure 4 presents the percentages of the treatments and their chronological trends. For the esophagus and stomach, endoscopic treatment increased over time, whereas the proportion of surgeries decreased gradually. Treatment of the colon and rectum was stable for 4 years. Although the cancer registry uses common items for all cancers, it codes trans‐arterial chemoembolization and radiofrequency ablation as others, making this category occupy a major proportion of all cases.

## 4 CONCLUSION

Statistical information is essential for clinical cancer research and policymaking processes. We summarize the essential information in this paper to help users of these statistics understand how these are made and what caution they need to take when interpreting the numbers. We hope that this information will enhance research and cancer control in this field.

## FUNDING INFORMATION

This work was funded by a grant‐in‐aid from the Ministry of Health, Labour, and Welfare.

## CONFLICT OF INTEREST STATEMENT

Y. Kurokawa is an Associate Editor of the *Annals of Gastroenterological Surgery*. T. Higashi was employed by National Cancer Center and was in charge of operating National Cancer Registry and Hospital‐based Cancer Registries.

## ETHICS STATEMENT

Approval of the research protocol: This study used only already publicly available information. As per the Japanese Ethics Guidelines for Human Subject Medical Research, such secondary studies do not require an ethics review.

Informed Consent: N/A.

Registry and the Registration No. of the study/trial: N/A.

Animal Studies: N/A.
